# Broken Promises to the People of Newark: A Historical Review of the Newark Uprising, the Newark Agreements, and Rutgers New Jersey Medical School’s Commitments to Newark

**DOI:** 10.3390/ijerph18042117

**Published:** 2021-02-22

**Authors:** Rosy C. Franklin, Ryan A. Behmer Hansen, Jean M. Pierce, Diomedes J. Tsitouras, Catherine A. Mazzola

**Affiliations:** 1 New Jersey Pediatric Neuroscience Institute, Morristown, NJ 07960, USA; rozyfranklin2011@gmail.com (R.C.F.); rah229@njms.rutgers.edu (R.A.B.H.); 2 Health Professionals and Allied Employees, Emerson, NJ 07630, USA; jpierce@hpae.org; 3 American Association of University Professors, Biomedical and Health Sciences of New Jersey, Newark, NJ 07103, USA; executive.director@aaupbhsnj.org; 4 Department of Neurosurgery, Rutgers New Jersey Medical School, Newark, NJ 07103, USA

**Keywords:** health workforce, workforce policy, health equity, racism, history, medicine, medical education

## Abstract

Many have referred to the coronavirus disease 2019 crisis and intertwined issues of structural racism as “twin pandemics”. As healthcare workers in Newark, New Jersey, a city heavily affected by the twin pandemics, we recognize that health workforce changes must be grounded in our community’s recent history. The objective of this essay is to briefly describe the relationship between organized medicine, state and local leaders, and the people of Newark. We begin with a discussion of Newark in the 1950s and 1960s: its people experienced poor socioeconomic conditions, terrible medical care, and the many sequelae of abhorrent racism. Plans to establish a New Jersey Medical School in Newark’s Central Ward also threatened to displace many residents from their homes. We then describe the Newark Agreements of 1968, which formalized a social contract between the state, business leaders, and people of Newark. In part, the Medical School committed to indefinitely promoting public health in Newark. We share progress towards this goal. Finally, we document key healthcare administrative decisions facing our community today. Stakeholder opinions are shared. We conclude that the Newark Agreements set an important standard for communities across the country. Creative solutions to healthcare policy may be realized through extensive community collaboration.

## 1. Introduction

Many have referred to the coronavirus disease 2019 (COVID-19) crisis and intertwined issues of global structural racism as “twin pandemics” [1,2,3]. Structural racism is defined as overarching systems, large-scale social forces, ideologies, organizations, and processes which interact to contribute to racial injustices and reinforce disparities [4]. The contemporary COVID-19 pandemic has illustrated the convergence of structural racism and health [5]. The United States medical community has renewed its interest in investigating the many effects of broader socioeconomic conditions on public health [6,7,8]. As medical students and physicians in Newark, NJ, we know that public health interventions are unlikely to prove effective if not informed by the past [9,10]. For Newark, we believe that decisions regarding healthcare administration and education must be grounded in our community’s recent history. In our recent history, a premier healthcare organization and training facility were established under conditions agreed upon with community members. Since their establishment, the school and hospital have remained integral pieces of the community and have taken significant steps to improve Newark’s public health. The “twin pandemics” are pressing issues which align to exacerbate structural inequalities. In response, changes to the healthcare system may be necessary, but the sociomedical history of Newark remains significant and informative, with important implications for healthcare decisions today.

During the 1950s and 1960s, the people of Newark experienced both poor socioeconomic conditions and the effects of abhorrent racism. Concurrently, plans to establish a New Jersey Medical School in Newark’s Central Ward threatened to displace many Newark residents from their homes. The historic Newark Agreements of 1968 (sometimes referred to as the Newark Accords), detail a compromise borne out of lengthy negotiations between the state, many business leaders, and Newark’s own community leaders. The legitimacy of Rutgers New Jersey Medical School (NJMS) and its primary teaching hospital, University Hospital (UH), is grounded in said Agreements. Moreover, development of Rutgers NJMS and UH in Newark’s Central ward established the two entities as critical cornerstones of healthcare provision and a place of work for some of Newark’s most impoverished minority groups. Today, ongoing administrative discussions regarding merging these entities with a private health system [11,12,13,14] threaten to erode decades of trust built between Newark’s people and its medical school. Some fear that valuable resources, indispensable personnel, and employment opportunities may be funneled from a still-ailing Newark community to New Jersey’s more affluent suburban communities [12,13,14,15]. The diversion of resources from programs meant to benefit Newark’s minority populations (including, but not limited to, communities of color and Hispanic communities) is not without precedent: in the 1960s, Mayor Hugh Addonizio diverted funding from the community-led United Community Corporation (UCC) [16]; and in 2018, Rutgers diverted inpatient pediatrics specialists and resources from UH [17].

Therefore, the objective of this essay is to briefly describe the relationship between organized medicine, state and local leaders, and the people of Newark, NJ. We begin with a description of how socioeconomic factors, combined with civic unrest, culminated in the Newark Uprisings of 1967. Discussions to establish a New Jersey Medical School, and the reasons for its location, are shared. The subsequent Newark Agreements and outcomes of said Agreements to present day are discussed. Finally, we consider today’s discourse about the future of Rutgers New Jersey Medical School and Newark’s UH.

This interdisciplinary work aims to emphasize that local health policy decisions are, and have always been, complex. By specifically describing the relationship between Newark’s medical school and its community, we hope that readers of all backgrounds will better appreciate the role that factors such as race, power, and politics play in determining the ultimate health status of a community. The presence and investment in medical training facilities in Newark have helped to curb sociomedical inequities. University Hospital is an essential safety net hospital in Newark. Readers may learn from Newark’s local history and apply relevant lessons to improve public health in their own communities. Accordingly, this manuscript is broken down into thematic sections. Readers may therefore gain a specific appreciation for each individual topic while simultaneously appreciating the intersecting themes present in each section.

## 2. Newark 1950–1967

To appreciate the magnitude of the Newark Agreements, it is imperative to first review the socioeconomic conditions in Newark, New Jersey in the 1950s and 1960s. In 1950, Newark’s population was 439,000 [18], where the Black community comprised 17% of that population [19]. Over the next two decades, social segregation was prevalent. Due to population growth as well as concurrent “white flight”, in which many white New Jersey residents left urban areas for suburban areas [20], the Black population in Newark reached 63% of the total by 1968 [19]. Unfortunately, the white flight of the 1950s and 1960s left Newark devoid of many of its manufacturing industries [19,20]; rates of unemployment were over 15% in the Black community [16]. Newark had high crime rates [16], and with one-third of the houses “substandard”, Newark had “the highest percentage of substandard housing for any city of comparable size” [19]. Forty-five percent of adults over the age of 25 had less than an eighth-grade education [19]. Unsurprisingly, these socioeconomic circumstances contributed to the community’s poor public health metrics: in the late 1960s, Newark had a high burden of substance use [16], sexually transmitted infections [16], the highest incidence of new tuberculosis cases in the country [19], as well as the highest maternal and infant mortality rates in the country [19]. While surrounding suburban towns had some of the best hospitals in the nation [19], Newark City Hospital was referred to as the “Slaughterhouse” or the “Butcherhouse” [19]. In 1969, Newark City Hospital had two nurses for every 39 patients; a rate dramatically lower than the reported state of New Jersey’s requirement of 1 nurse per 6–8 patients [19].

In response, the President Johnson administration sponsored an antipoverty program called the Model Cities Program in 1966 [16,19]. Model Cities gave federal funding to local governments with objectives to revitalize urban centers [16]. Newark leaders recognized the applicability of this program to their city; Newark’s 1967 Model Cities application stated that, “Today, poverty and the problems of racial transition are common to most older cities, especially in the Northeast. However, there are few cities anywhere in the nation where these and other problems extend so widely and cut so deeply as in Newark” [19]. Concurrently, sponsored by the federal Office of Economic Opportunity, Newark’s UCC was developed to help Newark’s poor. The UCC was a grassroots community action program aiming to “[enforce] housing codes and [train] minority citizens to qualify for high-paying union jobs in the construction industry” [16]. Given its unique element of community leadership, the UCC was a focal point for emerging Black power [16]. Election laws which prohibited individuals from voting unless they resided in Newark for at least six months, for example, had hindered many poor Black citizens from political engagement [19]. Funding from UCC helped Newark to sponsor a young Black man, Kenneth Gibson, to challenge the white Democratic Mayor Hugh Addonizio [16]. Gibson’s initial mayoral bid was unsuccessful [16]. Addonizio lobbied the federal government to divert funding from the UCC (for which funding stagnated by 1967 [16]) towards those programs in which he had more governmental control, such as the Model Cities program [16,21]. Unfortunately, the Model Cities program was poorly coordinated and has been cited to have contributed to Newark’s further socioeconomic breakdown [16]. Despite efforts from the federal government and local organizations to improve urban conditions, inequities in Newark persisted.

The social, economic, and medical injustices of the 1950s and 1960s culminated in members of Newark’s African American community feeling “despair, rage, impotence, racial pride, and the sense that police had a double standard… that condoned brutality toward Black Americans” [16]. Indeed, the civil rights movement of the 1960s was characterized by broader national unrest. Police violence sparked multiple rebellions across the country in the mid-1960s, including in: Watts, California; Detroit, Michigan; Tampa, Florida; Cincinnati, Ohio; Cleveland, Ohio; Chicago, Illinois; and Atlanta, Georgia [16,22]. As early as 1957, multiple African American newspapers and the Mayor’s Commission on Intergroup Relations in Newark documented “widespread mistreatment of Black Newarkers by police” [22]. Newark citizens, led by activist Amiri Baraka and the Congress of Racial Equity, joined their counterparts in Philadelphia and New York City in demanding for a civilian review of policing [22]. These activists led public demonstrations demanding a civilian review board for the majority-white Newark police force [22]. Mayor Addonizio sided with the Police Director and Newark Police Benevolent Association in obstructing these reviews [22].

## 3. Newark Uprising

On July 12, 1967, a Black Newark taxicab driver named John Smith was arrested for a traffic violation and was beaten by police [16,22,23]. The UCC and many community members rallied in support of John Smith, while the police arrived dressed in riot gear [16]. Violence broke out [16,23]. The National Guard was mobilized [16]. White-owned stores were looted [16,23]. From July 12–17, similar community uprisings occurred in Elizabeth, Englewood, Plainfield, and New Brunswick, New Jersey [16]. Twenty-four of the 26 total deaths were of Black civilians, most of whom were killed by police firearms [22]. The “typical” rioter was found to be “an individual who had resided in Newark for greater than ten years” [19]. As one individual stated, “They had taken all the land over on 12th Ave. by ‘eminent domain’. They said they were going to build townhouses but never did. We had no place to move, jobs, housing or schools” [16]. Even when the New Jersey state’s Select Commission on Civil Disorders recommended a Newark review board in the aftermath, Mayor Addonizio objected to its formation [22]. Notably, the President Johnson National Advisory Commission on Civil Disorders report stated that “white racism is essentially responsible for the explosive mixture [resulting in widespread national upset] which has been accumulating in our cities since the end of World War II” [20].

In many historical sources, these events have been termed the Newark Riots [16,21,24,25]. A minority of sources instead describe these events as the Newark Uprising or Rebellion [22,26]. Given the historical context, we believe the terms Uprising or Rebellion are more appropriate descriptors of the events in Newark in 1967, in comparison to the more inflammatory term “Riot.” Throughout the remainder of this manuscript, we hereby utilize the term “Uprising.”

There are many factors that are believed to have contributed to the Newark Uprising. These include racism, widespread political disenfranchisement and voter suppression, poor housing and landlords, unemployment and job discrimination, poor health conditions, poor schooling, the conflicting goals of Newark’s multiple anti-poverty programs and the ensuing funding cuts to the UCC, police brutality, and the passing over of selecting a Black community member for a Newark Board of Education position [16,19,20,23,26]. One final factor noted by Marin to contribute to the Newark Uprising was “the New Jersey state medical school’s move to Newark’s Central Ward” [16]. As Duhl and Steetle stated, the medical school issue “helped create the atmosphere in which only a spark was needed to kindle the riot fire” [19].

## 4. Medical School Plans

Concurrent with the social segregation, racism, widespread social unrest, poverty, and worsening public health status were developments to organize medicine in northern New Jersey. Nationally, concerns about physician shortages dominated discussions around physician workforce policy during the 1950s, 1960s, and early 1970s [27]. Federal initiatives during this time included construction grants to medical schools to bolster production of new physicians [27]. Leaders in North New Jersey, likewise, recognized the need to increase healthcare workforce training locally. Several developments in local organized medicine were implemented over the next few decades. Reasons for the developments included the provision of ideal workforce training, the provision of care for the poor, improvements to the economy, and (as some residents felt) “community control”.

In 1949, Jersey City Medical Center applied for a National Institute of Health (NIH) research training grant and, in response, the National Heart Advisory Council suggested a New Jersey medical school be established [21]. Medical schools formed after World War II were typically not built in impoverished urban areas [21]. The proposal to form a New Jersey medical school was supported by the state of New Jersey, the city of Jersey City, and the American Medical Association (AMA) [21], but it was challenged by Seton Hall College and the local Catholic Church, who proposed that a “Seton Hall Medical School” be erected in Jersey City instead [21]. Seton Hall Medical School was ultimately established in Jersey City in 1956, but it quickly developed financial difficulties, resulting in the state reclaiming its control in 1965 and renaming it “New Jersey College of Medicine and Dentistry (NJCMD)” [11,24]. Due to the Jersey City Mayor’s frequent interference with the medical school’s affairs, NJCMD planned to move from the city by the mid-1960s [21].

Discussions quickly began regarding NJCMD moving to either Madison, an affluent suburban town in northern New Jersey, or to Newark [19]. Although faculty favored relocation to Madison, there were conflicts with their local community hospital [16]. In comparison, as of 1962, Newark Mayor Addonizio had offered Newark City Hospital “to any medical school interested in taking it over” [19]. The Newark site was the preferred destination by Essex County’s 13-member delegation, as well as reportedly by many “medical, civic, educational, religious, business organizations, and municipalities” [19]. Addonizio saw the medical school as an opportunity to revitalize Newark [16], increase employment within the city [19], gain increased funding through the Model Cities Program [16], and create “one of the finest medical facilities in the country” with research laboratories and a new “University Hospital” [16]. He believed the patients in Newark would be ideal for medical students in training [16]. In turn, Addonizio promised NJCMD a total of 167 acres in Newark’s Central Ward, including those occupied by Newark City Hospital [21]. The area, which he declared “blighted”, would be claimed by eminent domain [16]. 

In response to these housing threats, two community organizations formed: the Newark Area Planning Association (NAPA) led by Yale law student Junius Williams, and the Committee Against Negro and Puerto Rican Removal (The Committee) led by Newark public school teacher Harry Wheeler [19] and chairman Louise Epperson [16]. Harry Wheeler stated, “the real reason for courting the medical school was that Addonizio wanted to disperse the Negro’s political power” [19]. Both NAPA and The Committee used legal and administrative tactics to fight the medical school proposal [19]. They acknowledged that medical school discussions lacked a plan for the relocation of residents displaced by NJCMD’s construction and did not include plans for the inclusion of city construction trade unions in building of the school [19]. In addition, there was a fear that “a new University Hospital… would be likely to exclude poor city residents” [21].

In June of 1967, immediately precipitating the Newark Uprising, NJCMD agreed to move to Newark [19]. Both the NAACP Legal Defense Fund and NAPA protested to the federal Department of Housing and Urban Development (HUD) that HUD’s relocation procedures were violated by the medical school plan [16]. HUD Undersecretary Robert Wood and Health, Education, and Welfare Undersecretary Wilbur J. Cohen called for community-wide negotiations [16]. Many believed the medical school was “being used as a pawn” in a broader struggle for community control [19]. One individual stated, “no school in the history of medical education has been created under such circumstances” [16].

The ensuing medical school relocation discussions have prompted many poignant reflections:

- The whole question of what is medical education came into play here. The College perceived the function of medical education in classical terms—providing an arena for the development of doctors…. The ills of the ghetto are viewed in nonmedical terms… the medical profession tends to feel that it is the only group competent to make decisions about medical education; all others… pose a threat to good education [19].
- Many medical schools are situated in or near inner-city slums, and indigent patients from these blighted areas traditionally have been important in the training of students [25].
- The College’s very existence now hinged on what had previously been peripheral to the interests of medical education: housing, employment, and citizen participation [19].

## 5. Newark Agreements/Accords

On 1 March 1968, after extensive local meetings and negotiations, the revolutionary Newark Agreements were signed [28], with amendments finalized on April 30 of the same year [16]. These Agreements are a historic social contract between the Newark community, the medical school, and governments at the local, state, and federal level [28]. Parties had agreed to commence construction of the academic medical center in the Central Ward of Newark [11,28]. The following points, as summarized in the excellent article by Marin, were included in the Agreements [16]:

1. NJCMD would reduce the size of the planned site from 167 to 57.9 acres.
2. NJCMD would utilize New Jersey state funding, as well as at least 2.5 million dollars independently fundraised, to “improve the quality of medical care [at Newark City Hospital] to a level equivalent to that expected of the teaching hospital that was to be built on its grounds”.
3. NJCMD would provide a comprehensive community health program for the residents of the Newark Central Ward, reviewed by a Newark Community Health Council.
4. Council responsibilities would include, to:
  - “develop a comprehensive health plan for Newark’s low-income community”;
  - “develop a comprehensive community mental health plan for Newark’s low-income community”;
  - “operate community health programs”;
  - “formulate and coordinate training programs in health services and professions”;
  - “assist the school in actively recruiting minority students, faculty, and professional staff”;
  - “work with the school to develop ‘career ladders’ for non-professionals in the health field”;
  - “periodically review the adequacy of community health services being provided by the school and make suggestions for change”.
5. Residents would be assured admission to the teaching hospital without bias.
6. A Community Housing Council would be formed to meet the needs of those displaced.
7. Minority group employment would be offered at the construction site, and NJCMD would include affirmative action clauses into contracts and subcontracts.
8. NJCMD would employ community residents in “as many of the 2600 jobs [expected to be produced]” as possible.
9. The Newark City Demonstration Agency would adhere to the regulations of the Model Cities Program.

## 6. After the Agreements

The Newark Agreements’ principles are binding and, therefore, success in fulfilling the aforementioned commitments has varied [21]. Initially, there were many challenges. In the immediate aftermath of both the Uprising and the Agreements, vigilante groups of white individuals formed in Newark under the guise of protecting their “families and homes” [19]. Many more white and middle-class Black individuals moved away from Newark permanently [16]. In 1970, borne out of accusations of poor-quality medical care at Newark City Hospital and the poor treatment of employees, Newark community members protested the hospital [16]; this culminated in resignation of the NJMS president. Then, in the winter of 1971, the community attempted to block construction of a teaching hospital over concerns that it would function as a white referral hospital; ultimately, then-Mayor Kenneth Gibson helped in deciding to construct a single hospital (UH) to meet the needs of all community members [16]. Although perhaps most significantly, the Newark Community Health Council failed in its mission due to supposed in-fighting among members [16]. The Council was replaced in 1971 by the Board of Concerned Citizens, created and governed by the NJMS Board of Trustees [16]. Authors have noted that “the mission of developing a comprehensive health program for the community was substantially lost” and, instead, the new Board served only as an ambassador between school and community [16].

In 1970, Governor William Cahill enacted legislation merging NJCMD into a broader “College of Medicine and Dentistry of New Jersey” (CMDNJ), and the medical school adopted the title of “New Jersey Medical School” (NJMS), which it still holds to this day [29]. By May 10, 1976, the Newark campus was completed, including the medical school, dental school, Community Mental Health Center, and primary teaching hospital and level-one trauma center for the entire state of New Jersey [28]. A 1977 conference held to assess the school’s progress in upholding the Newark Agreements found that: NJMS had invested substantially in Newark City Hospital [16]; the majority of the 2600 new jobs created were held by Newark community members [16]; citizen relocation agreements had been upheld [16]; and NJMS had begun to establish community health services, including a Family Health Center, preventative medicine and substance abuse programs, an ambulance service, and a CompreHealth health maintenance organization/ healthcare delivery system dedicated to Newark citizens [16]. In addition, NJMS had the largest enrollment of minority students of any medical school in the country, excluding two historically Black medical schools [16]. This fact remains true to date [11,13].

In 1981, the College of Medicine and Dentistry of New Jersey was reestablished as the “University of Medicine and Dentistry of New Jersey” (UMDNJ), of which NJMS was a part, making it “the largest freestanding public university of health sciences in the United States” [11,28]. Additionally, in the 1980s, the AIDS epidemic would take a significant toll on both Newark and its healthcare system [30]. By 1989, New Jersey ranked fourth in the nation in the number of reported AIDS cases [30]. It also had the highest percentage of women with HIV infection in the entire nation [30]. Newark was a major focus for infection. Today, New Jersey remains as “the epicenter of the HIV epidemic” [31]. Even early on, UMDNJ-NJMS physicians were at the forefront of the epidemic. Based on clinical experiences at UMDNJ-NJMS, a group led by Dr. James Oleske published a landmark study on immune deficiency syndrome in children, which for the first time drew attention to the fact that AIDS could affect children as well [32].

In 1994, the American Association of Medical Colleges (AAMC) awarded an Outstanding Community Service Award to the medical school at UMDNJ [16]. UH’s mission as a valued community resource has been emphasized throughout its establishment, including in a 2010 New Jersey Higher Education Task Force report [11]. Per this report, UH and the medical school campus have played a fundamental role in Newark’s community, economic, and cultural revitalization [11]. UH cares for the most uninsured patients in the state of New Jersey, and is the only public acute-care hospital in the state [14]. The report specifically notes that they believe continued additions to the Newark medical campus “[build] on and [enhance] the historic Newark Agreements” [11]. Several NJMS-sponsored community outreach organizations do important work in Newark, as briefly reviewed later in this article. NJMS is the oldest school of medicine in the state of New Jersey, and today it receives the most NIH funding for basic and clinical sciences out of all schools in the state [13].

However, rumblings of community discontent have persisted: a notable piece in late 1987 in the Newark Star-Ledger, penned by Joan Whitlow, reasserted that the school had engaged in discriminatory hiring practices, as well as failed to further increase minority student enrollment at the medical school [16]. In 2013, oversight of the medical school was restructured such that UH became an independent NJ state entity, while NJMS remained within Rutgers Biomedical and Health Sciences (RBHS) [33].

In the last few years, there have been several notable changes at UH. In early 2018, UH submitted a proposal to the New Jersey Department of Health (DOH) to reduce inpatient beds for children from twenty-three to four due to low patient volume [14,34]. Specialist physicians in pediatric trauma and resident pediatric physicians would be transferred to a different hospital, Newark Beth Israel [34]. Given that UH is the only Level 1 trauma center in New Jersey, many physicians and nurses stated that reducing pediatric care in this hospital would be detrimental for families in Newark [34]. In fact, some feared that the move would jeopardize UH’s entire status as a Level 1 trauma center [14,34]. As Dr. James Oleske, professor of pediatrics, stated at the time, “this is a death blow for our medical school’s commitment for that segment of the population… To abandon the Central Ward and take pediatrics away from University Hospital is a terrible mistake” [34]. Dean of Rutgers New Jersey Medical School and Chair of University Hospital’s Board of Trustees, Robert Johnson, stated that the hospital “wasn’t built to have pediatrics in it” and that he has attempted to move pediatrics out of UH since the late 1990s [34]. As of July 2018, it was reported that UH withdrew this DOH proposal [35]. New Jersey Governor Philip Murphy ordered the DOH to appoint a monitor to review this situation with UH. The ensuing 2018 report stated that, “[UH] started to decrease its pediatric bed complement without the documented approval of the State… in seeming contradiction to the Restructuring Act and the 1968 [Newark] Agreement” [17]. Indeed, this decision was made without even alerting Newark’s mayor [14]. To the best of our knowledge, the inpatient pediatrics unit at UH remains significantly downsized.

In late 2018, the UH CEO John N. Kastanis resigned amid both calls from the mayor and scrutiny from the DOH regarding an *Acinetobacter baumannii* bacterial outbreak at UH, ending a short tenure at a hospital already juggling significant changes [36,37]. The outbreak in question may have claimed the lives of three infants in the neonatal intensive care unit [36]. Just half a year later, in 2019, Rutgers University President Robert L. Barchi announced that he too would step down after the upcoming school year [38]. This resignation ended a seven-year tenure in which Rutgers both joined the Big Ten Conference and completed the largest higher education merger in American history [38].

## 7. Newark Today

It is critical to acknowledge that economic stability, neighborhood and physical environment, education, food, community and social context, and healthcare system are all known social determinants of health [39]. In other words, the broader lives and lived experiences of those in Newark impacts the ultimate health status of those individuals. As of 2017, Newark has a population of 285,154 individuals [40], representing growth of 4.2% since 2000 [40]. Median age is approximately 34 years [39,40], and 51% are female [39]. The racial demographics have changed since the 1960s: today, approximately 89% of the population are racial/ ethnic minorities, with 48.6% Black and 35.6% Hispanic/ Latino [39]. In addition, 30.6% of the city’s population is foreign-born [39], 54.2% speak English only, and 30.9% speak Spanish [39].

It is also critical to acknowledge that the modern socioeconomic state of Newark was largely determined decades ago; systems of structural racism have helped to translate poor conditions for minority groups during the 1950s and 1960s into poor conditions for minority groups now. The estimated poverty rate in Newark is generally 29% and, among children, increases to 39.5% [39]. Newark had an estimated median household income in 2017 of USD 35,167 (compared to the New Jersey average of USD 80,088) [40]. Black and Hispanic or Latino individuals are more likely than their white Newark counterparts to live in poverty [40]. Only 16% have a Bachelor’s degree or higher (compared to 37.6% of New Jersey residents) [39]. Accordingly, the unemployment rate, as of March 2019, was 6.7% [39]. Nearly 28% of Newark households have utilized Supplemental Nutrition Assistance Program (SNAP) benefits in the past 12 months [39]. The most common industries in Newark as of 2017 are healthcare (11.2%), followed by construction (9.6%) and accommodation and food services (7.6%) [40]. Notably, 18.4% of women in Newark work in healthcare [40].

Although the demographics of Newark’s population have shifted, persistent socioeconomic disadvantages continue to influence a host of poor public health outcomes. Today, an estimated 39.0% of Newark adults have hypertension (vs. 28.6% for NJ overall), 15.7% have diabetes (8.7% NJ), 8.4% have chronic obstructive pulmonary disease (5.1% NJ), 7.6% have coronary artery disease (5.8% NJ), 4.6% have had strokes (2.2% NJ), and 25.0% have lost all of their teeth (13.3% NJ) [39]. The percentage of Newark women giving birth in 2015 without any prenatal care was 3.1%, compared to 1.4% for New Jersey overall [39]. The overall infant mortality rate is 11.6% (vs. 4.4% for NJ overall), with a rate of 15.1% for Black infants (8.7% NJ) [39]. Although only about 3.1% of New Jersey’s population resides in Newark, 17.4% of New Jersey’s primary and secondary syphilis cases are in Newark [39]. These issues are partially because 28.9% of Newark individuals aged 18–64 lack health insurance (compared to 15.7% in New Jersey, overall) [39]. Although passage of the Patient Protection and Affordable Care Act in 2010 increased rates of covered individuals in Newark from 71.9% to 81.9% in 2015, there are still approximately 50,000 individuals lacking insurance [39].

It is critical to understand the concept of social determinants of health, particularly during the global COVID-19 pandemic. Authors have written extensively about the role of structural racism in exacerbating existing health disparities in the time of COVID-19 [5]. We wish to emphasize that the people of Newark have similarly been impacted [41,42]. Since the beginning of the pandemic, activists warned that people from Black and brown communities in New Jersey would disproportionately be burdened by COVID-related morbidity and mortality [43] and, unfortunately, they were correct [41,42], emphasized in the sobering figure created by the New Jersey Policy Perspective in October 2020 [42]. This new health disparity is a product of Newark having disproportionately fewer medical and economic resources than surrounding wealthy communities in Newark [44]. In October, USA Today did an entire expose on the role of structural racism and health disparities in ravaging Black and Brown communities in Essex County (Newark’s county) during the COVID pandemic [45]. We encourage all readers to review this piece. It explains how during the first wave of the COVID pandemic, “Essex County was among the top 10 in the country for its death rate”. In addition, Newark’s Mayor Ras Baraka emphasized that many Newark citizens do not have the luxury to isolate in a basement or attic, apart from their family members, during quarantine [44]. As a result, many Newarkers have been contracting COVID within their own homes [44]. Even with the rollout of COVID vaccines, offered to various community members at Newark’s own New Jersey Medical School campus to date, Mayor Baraka and others have expressed fear that the people of Newark may lack trust in the medical system to such an extent that they are unwilling to receive the vaccine [44].

Violence is also a known public health issue [18]. A 2014 Department of Justice investigation of the Newark Police Department found widespread discriminatory policing and excessive use of force [22]. In 2015, Newark Mayor Ras Baraka signed an executive order to establish the Civilian Complaint Review Board (CCRB) to evaluate the Newark Police Department [22]. Notably, civil rights activists in Newark have persistently demanded for a CCRB since the 1970s [22]; the issue was frequently revisited, politicized, and then opposed by key political leaders until 2015 [22]. Although the crime index in Newark has decreased from 2013 to 2018 [40], issues of police brutality still permeate civic life in Newark as well as in cities across America. Police brutality, which disproportionately affects people of color, may manifest as physical, sexual, and emotional abuse; modulating stress levels and contributing to the development of many downstream acute and chronic health issues [22]. These experiences of stress may be carried forward throughout generations [22]; a sobering reality for families in Newark. To this day, activists and allies continue to advocate for health equity, anti-racism, and safety in their communities. On just one 2020 summer day in Newark, for example, over 12,000 individuals participated in a peaceful protest of police brutality [23]. There is, however, more work to be done.

## 8. Medical School Today

Certainly, the primary purpose of the medical school is to train tomorrow’s physicians. However, it is apparent that the school’s unique founding, out of extensive negotiations with its community, has had an enduring impact on the school’s values. Below, we present some of the ways in which New Jersey Medical School’s student body have demonstrated a deep understanding of the school’s mission to address the public health needs of its community. The relative success of these efforts is beyond the scope of this manuscript; we encourage readers to review the specific published manuscripts briefly cited below. 

One of the major social determinants of health is the healthcare system [39]. Key system factors include insurance coverage, physician availability and cultural humility, available language services, and quality of care [39]. Rutgers New Jersey Medical School currently has many efforts to address these factors as well as other social determinants of health; it is nationally recognized for its community collaboration [11,13]. It is our privilege to highlight a mere select few civic engagement efforts spearheaded by NJMS’ medical students and published in academic journals.

Briefly, the African-American Brain Health Initiative is a university-community partnership which “combines community engagement, education and training, and brain health research” [46]. It aims to promote brain health and participation in brain-related research initiatives among elderly African Americans in Newark [46]. The Ironbound Initiative is a student-led group at Rutgers NJMS, in conjunction with public and private community organization partnerships, which works to build trust between the healthcare system and the growing Latino community [47]. They have partnered with Mantena Global Care, a Brazilian community organization in Newark, to disseminate COVID-19-related information [47].

Benefits of New Jersey Medical School community endeavors extend beyond the provision of healthcare and connection to resources for the local community; they serve as opportunities for medical students to learn from the people they hope to serve. MiniMed is an outreach program designed to “empower the powerless to communicate more effectively with clinicians” via providing opportunities for medical students to interact with people from disadvantaged social groups in a non-threatening context [48]. For example, medical students have prepared and delivered lectures to inmates at Kintock Group facilities [48]. Through partnering with the Kintock Group and Newark Renaissance House, which is a nonprofit residential therapeutic community to assist chemically dependent women and children, participating medical students may become more familiarized with the circumstances, social programs, and healthcare needs for these patients [48]. Other ongoing school efforts are described online [49,50].

## 9. Medical School Tomorrow

Presently, Rutgers Biomedical and Health Sciences includes two separate medical schools, as well as colleges of nursing, pharmacy, dentistry, and other health sciences [12,33]. A proposal to merge the medical schools, which have campuses in Newark (NJMS) and New Brunswick (Robert Wood Johnson Medical School), has not yet been decided publicly [12]. However, Chancellor Brian Strom, hired to oversee RBHS in 2013, has proposed to combine the medical schools in the future, believing “a single accredited institution—stretched over two urban campuses—would be better for students and attract more research dollars” [12]. Currently, Strom serves as Chancellor of both medical schools, is on the board of UH, and is also on the board of the New Jersey Barnabas Health System [51]. Rutgers states the arrangement with RWJ Barnabas Health “is designed to create a higher quality, more sustainable health care system throughout northern and central New Jersey” [12]. Additionally, former Governor Chris Christie, who has acted as a lobbyist for several NJ hospitals during the pandemic, has been hired by RWJ as a consultant [52].

Despite Chancellor Strom’s vision, several notable groups have expressed concern about the future in Newark. Indeed, UH is the primary teaching hospital for NJMS. These concerns have come in the form of letters by Senator Ron Rice, on behalf of members of the NJ Legislative Black Caucus, and from various health care unions (Health Professionals and Allied Employees; Communications Workers of America Local 1031; American Association of University Professors Biomedical Health Science of New Jersey) and sent to the Rutgers University President and to New Jersey Governor Phil Murphy [12,14]. They have cautioned that “Rutgers’ partnership with a massive private hospital system and efforts to reorganize its two medical schools will drain staff and other resources from the urban hospital, which serves many vulnerable patients in Newark and functions as the state’s only public acute-care facility” [12]. Indeed, some fear that UH will lose its status as an academic teaching center or close entirely [12]. It has been asserted that Rutgers plans are being developed “without sufficient public and stakeholder input” [12,15]. Over 1500 faculty from both medical schools have publicly opposed any such medical school merger at this time [15,51]. Faculty have contended that even the Council appointed to review the question of a medical school merger [53] does not sufficiently represent elected faculty leaders from either medical school [15].

Newark Mayor Ras Baraka strongly opposes the merger of the two healthcare systems, characterizing it as “an unregulated and premeditated takeover that will leave Newark residents without critical resources” [13,51]. In an open letter published on NJ.com, he stated this is “one of the ways that systemic racism rears its ugly head” [13]. He fears that Newark’s residents will lose access to quality healthcare at UH and, therefore, current health disparities will be exacerbated. Baraka notes that these discussions violate the Newark Agreements [13]. Proper needs assessments, review for compliance with state laws and regulations, and public hearings—per the Mayor and others—have not been performed [13,14,51]. As he states, “somehow, when it affects those that are disadvantaged, there can be found a way to bypass process and procedure altogether” [13]. Of note, CWA president John Rose has pointed out that “many of [RBHS’s] other facilities are in suburban areas that tend to generate more lucrative reimbursements, compared with urban hospitals that care for high numbers of patients on Medicaid” [12].

Publicly, a spokesperson for RWJBarnabas Health explained that “RWJBarnabas Health respects and is sensitive to the unique histories of the medical schools, University Hospital and the City of Newark, and always seeks to demonstrate that respect. We are excited about the opportunities for enhancing the health of all New Jersey residents with our plans, and nothing within RWJBH’s relationship with Rutgers University negates either the terms or the spirit of the Newark Agreements” [12]. Rutgers states that UH and “public health in Newark remain priorities for the university as it evolves” [12]. Neal Buccino, associate director of media relations for Rutgers University states that “Rutgers is and remains fully committed to University Hospital and Newark, and no future organizational changes, should they occur, will change that” [12]. They have shared that they “understand the mayor’s passion” [51]. 

While reflecting on these current discussions, we are reminded of a quote by New Jersey Governor Richard J. Hughes upon creation of the Newark Agreements. At that time, he stated that, “the lengthy negotiations were designed to ensure that major advances in housing and relocation, community health services, training and employment opportunities, and community participation will in fact occur when the [school] comes to Newark… [representing] a pattern of constructive social action that brings together, as full equals, public official and private citizen, Black and white—a pattern that nourishes and dignifies everyone associated with it and that portends only good for Newark” [16].

It appears critical that New Jersey Medical School’s future is handled with similar care. The ongoing presence of Rutgers NJMS at 185 South Orange Avenue, Newark, will continue to benefit some of the least privileged members of our society. Continued investment in UH is necessary for it to remain a place of employment, teaching, and healing for Newark’s citizens; a 2010 report even stated that, “Rutgers needs to continue to expand not only its academic programming in Newark, but must commit to enhancing an ongoing residential and community presence in the city” [11]. These facts are emphasized by the unprecedented challenges posed by the current COVID-19 pandemic [6,54,55]. Achieving health equity for New Jersey’s most vulnerable patients must be a top priority. Striving toward success in this regard requires careful and strategic organization of New Jersey’s health workforce. The historic agreements established in the Newark Accords should not be undermined. Accordingly, when considering any business decisions to relocate any faculty, staff, and students from the Newark campus, we believe engaging in a similar approach as the 1968 Newark Agreements is in order. Communication is paramount. Our past teaches us that more creative, and ultimately more mutually beneficial solutions, will be generated by doing so.

## 10. Conclusions

We believe that it is imperative to know our community’s recent history. Elsewhere we have written about the ethical obligation of medical schools, generally, to engage with their communities to improve public health [49]. This article emphasizes that, among medical schools, Rutgers New Jersey Medical School’s commitment to Newark is meaningfully unique. Born out of widespread socioeconomic injustices, Rutgers NJMS have committed, indefinitely, to actively promote public health in Newark, codified explicitly in the historic Newark Agreements. We know of no other social contract like this one.

Today’s many instances of police brutality against Black and brown people, combined with the global COVID-19 pandemic that disproportionately harms and kills racial and ethnic minorities [7,8,54,55], are reminiscent of the devastation experienced by Newark citizens back in the 1960s. Additionally, current considerations to effectively re-distribute resources from RBHS’ Newark medical campus to more affluent hospitals eerily echo Mayor Addonizio’s redistribution of funding from the UCC to programs in which he could exert more control in the 1960s.

The Agreements set an important standard for communities across the country: hospital health policy decision-making may be a collaborative endeavor. Accordingly, we believe it is imperative that members of Rutgers NJMS, Rutgers RBHS, and affiliate faculty, staff, residents, and students review Newark’s recent history. These members must realign themselves with the commitments made by our own predecessors in the Newark Accords. When considering novel business ventures to potentially alter hospital structures, reorganize the health workforce, or to merge medical schools, all must remember that NJMS as we know it exists only because of extensive negotiations with the people of Newark. The ensuing Agreements lack an expiration date.

Of course, other works have more substantially explored the recent history of our community [19], as well as documented the Newark Accords [16] and the development of New Jersey Medical School [21]. Indeed, Robert Curvin’s book *Inside Newark* provides a comprehensive, authoritative analysis of recent sociopolitical developments in our city [26]. The goal of this interdisciplinary manuscript, therefore, is to connect the historical inequities in Newark to the current health care policy discussions in Newark. We found that it was impossible to adequately discuss historical health policy in Newark without simultaneously documenting efforts to achieve racial justice in Newark. Ultimately, we know that Newark’s story emphasizes the complex interplay between race, politics, and medicine in shaping a community’s past, present, and future public health. We believe the themes of this discussion are universal: Public health matters, local history matters, and creative health policy solutions may be implemented with improved communication. An international audience may find this piece thought-provoking. Hopefully, readers of all backgrounds will apply lessons described in this brief review to pursue health equity within their own communities.

## Data Availability

Not applicable.
